# Integrative Rosacea Treatment: Combination of a Low Crosslinked Injectable Hyaluronic Acid Filler With Standard Therapeutical Interventions—An International Real World Case Series

**DOI:** 10.1111/jocd.70199

**Published:** 2025-04-23

**Authors:** Ilaria Proietti, Sadiye Kus, Emanuele Amore, Francesca Svara, Chiara Battilotti, Concetta Potenza, Patricia Ogilvie

**Affiliations:** ^1^ Dermatology Unit Daniele Innocenzi, A. Fiorini Hospital Terracina Italy; ^2^ From the Private Dermatology Practice Istanbul Turkey; ^3^ Dermatology Unit, Department of Clinical Internal, Anesthesiological and Cardiovascular Science University of La Sapienza Rome Italy; ^4^ Skin Concept Munich Germany

**Keywords:** filler, hyaluronic acid, rosacea

## 1 Introduction

Rosacea is a chronic inflammatory skin disease that primarily affects the central area of the face, causing erythema, telangiectasia, swelling, and often small papules resembling acne. It has a multifactorial etiology with genetic factors, environmental triggers, and abnormalities in blood vessels and the immune system playing a pivotal role. The milder form, known as erythematous rosacea, commonly manifests with erythema, transient redness, individual telangiectasias, alongside hypersensitivity symptoms and cosmetic intolerance [1]. The burden of disease of rosacea and it's implication on patient's quality of life is a frequently underestimated aspect of this common skin disease [2]. Primary treatments commonly include topical therapies and oral antibiotics, alongside the avoidance of triggering factors [3]. Nonetheless, these therapies frequently fail to sustain a lasting response. In recent years, innovative techniques have emerged with the aim of achieving long‐term cosmetic improvements. In this context, laser and light sources, such as the long‐pulsed neodymium‐doped yttrium aluminum garnet laser (Nd:YAG), intense pulsed light (IPL), potassium titanyl phosphate (KTP) laser, and pulsed dye laser (PDL) therapy, have been proposed due to their effectiveness in treating telangiectasia and erythematous lesions [4]. Moreover, evidence suggests Hyaluronic acid (HA) as a potential strategy in the treatment of inflammatory skin conditions, including rosacea, either alone or in combination with other interventions [5].

## 2 Cases Presentation

**FIGURE 1:**
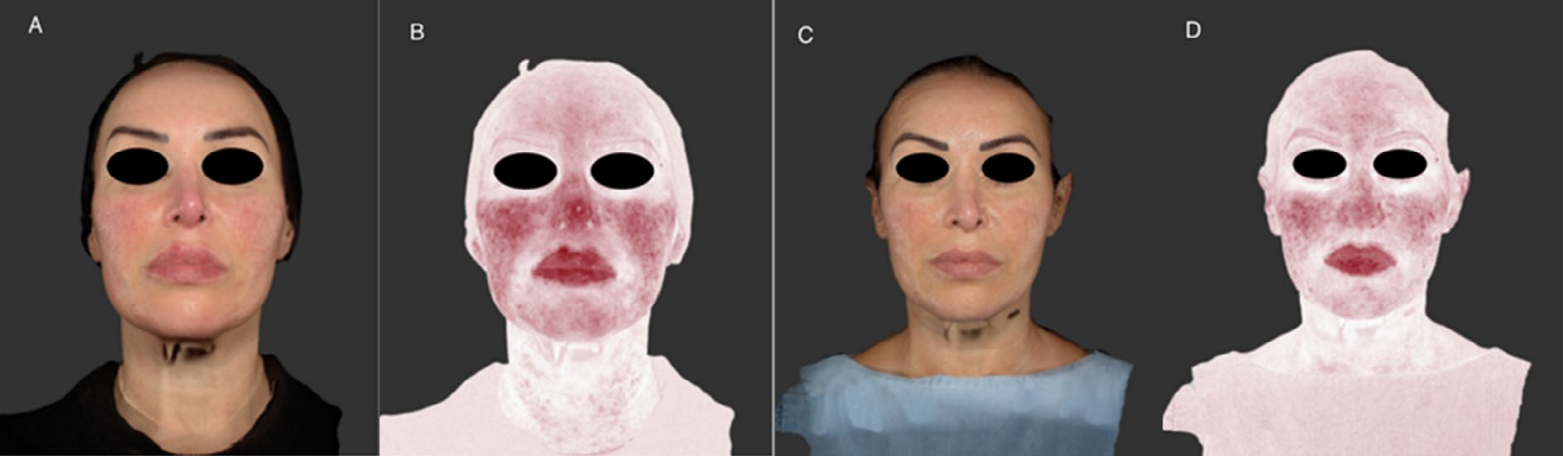
VECTRA evaluation of skin parameters (spots and red areas) at T0 (A, B) and after 4 months from treatments (C, D).

Case 1: A 52‐year‐old woman, Fitzpatrick scale II, who presented in March 2023 with erythematous rosacea previously treated with doxycycline and topical ivermectin, yielding temporary benefit. Upon clinical evaluation, erythema and localized telangiectasia were observed in the malar and nasal regions associated with episodic burning sensation and cosmetic intolerance. The patient underwent a Nd:YAG session at 6 joules followed by an IPL session at 14 joules 1 month later, in April 2023. Finally, in June 2023, micro‐injections of Vycross HA filler (VYC‐12) were performed in the reticular dermis of the malar and nasolabial regions, totaling 1 mL per side. After 4 months from HA injection, improvement in skin quality and reduction of erythema were observed. Standardized photographs were obtained at baseline and at the four‐month follow‐up visit using the VectraH1camera system (CanfieldScientific Inc., Fairfield, New Jersey) (Figure 1).

**FIGURE 2:**
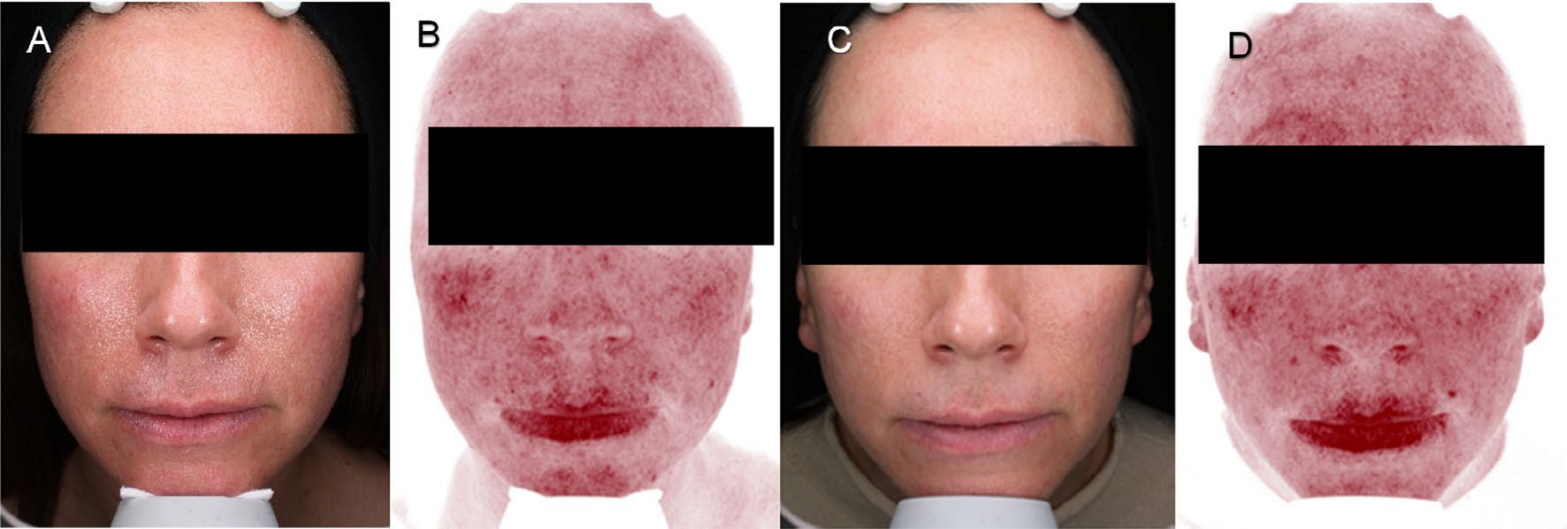
VISIA evaluation of skin parameters (spots and red areas) at T0 (A, B) and after 6 months from VYC12 treatments (C, D).

Case 2: A 38‐year‐old patient with Fitzpatrick scale II presented in June 2024 with moderate erythema, dryness, and sensitivity present for 4 years. While waiting for winter to introduce vascular laser treatment, the patient underwent one session of VYC‐12 L (1 cc per cheek), with microdroplets injected into the dermis to enhance skin hydration and prime the skin. Alongside, she was prescribed topical ivermectin and a tea tree oil facial shampoo. six months later, she received 2 sessions of 585 nm solid state laser treatment (DenaVe DEKA). Standardized photographs were taken at baseline and at the 8th month follow‐up using the Visia system (Canfield Scientific), confirming a reduction in erythema and improvement in texture and radiance (Figure 2).

**FIGURE 3:**
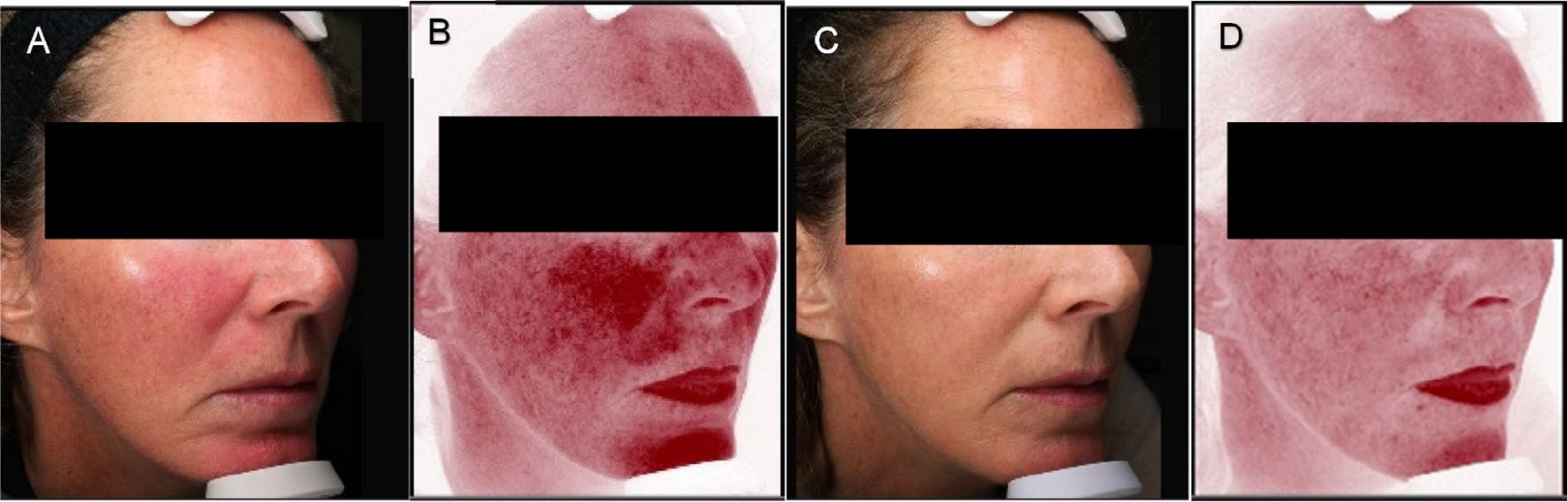
VISIA evaluation of skin parameters (spots and red areas) at T0 (A, B) and after 6 months from VYC12 treatments (C, D).

Case 3: A 52‐year‐old patient with Fitzpatrick scale II, who presented in October 2022 with mild erythema. The patient underwent a combination of treatments, starting with one session of IPL (560 nm Quantum SR Lumenis) at a fluence of 23 J/cm^2^ on day 0, aimed at targeting skin redness and improving overall skin texture. Two weeks post‐IPL, the patient received one session of VYC‐12 L (1 cc per cheek), with microdroplets injected into the dermis to enhance skin hydration and smoothness. Following these interventions, the patient did not require any further procedures, and a stabilizing therapy regimen was implemented. This included monthly in‐office glycolic acid peeling sessions (alpha/beta), utilizing a combination of glycolic acid to exfoliate and enhance skin renewal. At the two‐month follow‐up, the patient reported noticeable improvements in skin texture and a reduction in redness, with visible results on Visia imaging. Standardized photographs were taken at baseline and at the two‐month follow‐up using the Visia system (Canfield Scientific Inc., Fairfield, New Jersey), confirming the positive outcomes (Figure 3).

## 3 Discussion

HA is a naturally occurring polymer synthesized within the extracellular matrix (ECM) by connective tissue cells. With properties including hydration, cell regeneration promotion, elasticity enhancement, biocompatibility, and biodegradability, HA is widely utilized in cosmetics and medical applications [6]. The combination of energy‐based devices (EBD) and HA in rosacea treatment offers several advantages. IPL and Nd:YAG laser target inflammation and dilated blood vessels, reducing redness and telangiectasia, while stimulating collagen production, improving skin elasticity, and minimizing wrinkles, enlarged pores, and acne scars [4]. HA injections performed several months after or before energy‐based device treatment provide hydration, texture improvement, and reduced inflammation [7], reinforcing the skin barrier and enhancing elasticity. The underlying mechanism of HA in reducing inflammation in rosacea likely involves its ability to modulate the skin's extracellular matrix, minimize post‐laser dryness, and optimize overall outcomes. Moreover, an increasing body of evidence shows that native and low cross‐linked HA provides anti‐oxidative and anti‐inflammatory properties, potentially explaining the synergistic effects of injection of HA with other established treatment modalities in the treatment of rosacea [8]. In the presented case, filler injections were well tolerated and did not lead to any adverse effects, contributing to reducing inflammation and increasing skin quality [9]. Our experience suggests that HA injections, specifically, low concentration, low crosslinked HA preparations, are not only not contraindicated but also effective and safe in the management of mild to moderate cutaneous inflammatory conditions, highlighting its potential role in combination therapies.

Clinical studies, analyzing the correlation of efficacy of low crosslinked HA facial treatment and psychological impact, have shown a noteworthy discrepancy: patient reported outcomes (PRO's), evaluating the psychological impact of treatment based on validated scales, showed a very high and long‐sustained multi‐layered patient satisfaction that did not parallel the improvement of skin texture and roughness. This indicates that sustained biological effects of low crosslinked HA leading to improved moisturization and possibly also anti‐inflammatory effects are the main drivers of patient satisfaction [10].

## 4 Conclusion

In this case series, we observed that the use of injectable HA appears to effectively stabilize and enhance the results achieved through established interventions such as laser treatments, EBD, and chemical peels. While the precise mechanism of action remains unclear, several potential explanations can be considered. These may include anti‐inflammatory effects, an optical “injectable camouflage” that improves skin appearance, and the presence of anti‐angiogenetic factors that may contribute to long‐term skin quality improvement. Further research is needed to better understand these mechanisms and fully elucidate the role of injectable HA in combination with other aesthetic treatments.

Treatments performed by Dr. I.P. (Figure 1), by Dr. S.K. (Figure 2), and by Dr. P.O. (Figure 3).

## Author Contributions

All authors were responsible for the concept and design of the study, collection and collation of data, analysis, and interpretation of data, writing an article, reviewing this article, final review of this article, and graphics performance.

## Ethics Statement

This study was reviewed by the local institutional review board (Ethics Committee of the Medical University Sapienza: Approval number US2345). Written consent is given by the patient.

## Conflicts of Interest

The authors declare no conflicts of interest.

## Data Availability

The data that support the findings of this study are available from the corresponding author upon reasonable request.
